# One-stage En bloc resection of thoracic spinal chondrosarcoma with huge paravertebral mass through the single posterior approach by dissociate longissimus thoracis

**DOI:** 10.3389/fsurg.2022.844611

**Published:** 2022-08-17

**Authors:** Wei Xu, Chen Ye, Dan Zhang, Peng Wang, Haifeng Wei, Xinghai Yang, Jianru Xiao

**Affiliations:** ^1^ Department of Orthopedic Oncology, Changzheng Hospital, Second Military Medical University, Shanghai, China; ^2^ Department of Radiology, Changzheng Hospital, Second Military Medical University, Shanghai, China

**Keywords:** *En bloc*, CHS, one-stage, approach, prognosis

## Abstract

**Study design:**

Retrospective case series.

**Objective:**

To describe the technique details and therapeutic outcomes of 3-D printing model-guided *en bloc* resection of chondrosarcoma (CHS) with huge paravertebral mass *via* the combined posterior median and Wiltse approach.

**Summary of background data:**

Total *en bloc* spondylectomy (TES) technique is conventionally based on the single posterior approach or combined anterior-posterior approach. However, the single posterior approach imposes a high technical demand on the surgeon due to the narrow field of vision, limited surgical space and the delicate spinal cord, while the combined anterior-posterior approach not only requires greater patient tolerance but is time consuming and runs the risk of more blood loss and injury to the visceral pleura and large blood vessels during surgery. In addition, it is difficult to completely remove the thoracic CHS with paravertebral mass through simple *en bloc* resection when it involves the aorta, vena cava, costa and lung.

**Material and methods:**

Between August 2010 and January 2016, we performed a retrospective study to evaluate the clinical characteristics and outcomes of *en bloc* resection of thoracic spinal CHS with paravertebral mass through the combined posterior median and Wiltse approach. Postoperative recurrence-free survival (RFS) and overall survival (OS) were estimated by the Kaplan-Meier method. P values less than 0.05 were considered statistically significant.

**Results:**

Altogether 15 patients received *en bloc* resection of thoracic spinal CHS with paravertebral mass through the combined posterior median and Wiltse approach. The mean age of these patients was 37.0 ± 12.8 years (median 36; range 15–64). This combination approach provided more extensive exposure and wider marginal resection of the tumor within a mean operation duration of 288 ± 96 min (median 280; range 140–480) and mean intraoperative blood loss of 1,966 ± 830 ml (median 2,000; range 300–3,000). Of the 15 patients, 5 experienced local recurrence of the disease; the mean time from surgery to recurrence was 22 ± 9.85 months (median 17, range 13–35). RFS in patients with recurrent CHS was significantly lower than that in patients with primary CHS on admission (*p* = 0.05).

**Conclusions:**

The combined posterior median and Wiltse approach is a technically viable option for *en bloc* resection of thoracic spinal CHS with huge paravertebral mass, and can give a favorable local control of CHS.

**Level of evidence:**

Level V.

## Introduction

Chondrosarcoma (CHS) is the second most common primary malignant bone tumor, accounting for about 25% of all primary malignant bone tumors (1, 2). It can be sub-classified as primary malignant bone tumors, or secondary malignant transformation of an underlying enchondroma or osteochondroma (3). It usually involves bones of the pelvic girdle, shoulder, and the proximal end of the femur and humerus (4, 5). But spinal CHS is a rare occurrence (6–8). Surgical resection remains the mainstay of treatment for CHS, knowing that it is insensitive to either radiotherapy or chemotherapy (1). Due to the extremely high rate of local recurrence after translesional excision, the goal of surgical treatment demands *en bloc* resection of the tumor with wide margins. Boriani et al reported that the local control rate was 82% in cases receiving *en bloc* resection vs. 0% in cases receiving intralesional excision in the mobile spine (9).

Total *en bloc* spondylectomy (TES) for thoracic spine malignant tumors is well developed and widely performed (10–12). However, it is difficult to *en bloc* resect thoracic CHS with paravertebral mass involving the aorta, vena cava, costa and lung.

In this study, we present a series of thoracic CHS cases with paravertebral mass, describe the surgical procedure and effects by the single posterior approach by dissociate longissimus thoracis.

## Materials and methods

### Study design and patients

A retrospective study was performed to evaluate the clinical characteristics and outcomes of *en bloc* resection of thoracic spinal CHS with a paravertebral mass. The final diagnosis of CHS was decided by postoperative pathology. Briefly, the tumor specimens were fixed in 10% formalin, paraffin embedded, sliced into sections, stained with standard hematoxylin and eosin (HE), and finally evaluated by a senior pathologist. The inclusion criteria were as follows: (1) patients with pathologically diagnosed CHS; (2) patients whose CHS involved the thoracic spine with paravertebral mass; (3) patients who received En-bloc resection of the tumor; and (4) the surgical procedures were performed by the same team of spinal tumor surgery.

Between August 2010 and January 2016, 15 patients with primary or recurrent thoracic spinal CHS with a paravertebral mass received *en bloc* surgical resection in our center. The tumors were staged by the Enneking and WBB staging system. The neurological function was recorded according to the Frankel score system. Postoperative adjuvant radiotherapy (PAR) was recommended to all included patients. The prescribed dose was 45–50 Gy. After a comprehensive and detailed explanation to the patient and family about the risks, benefits and costs of the radiotherapy, the patient and family decided to either follow or not follow the recommendation for radiotherapy. All the surgical procedures were performed through a posterior approach by a senior surgeon (Jianru Xiao)

### Surgical procedures

The patient was intubated in a prone position. X-ray was used to confirm the correct segments. A midline skin incision extending two or three vertebrae above and below the involved segments was made over the spinous processes. The paraspinal muscles were dissected to expose the posterior osseous elements of the spine. Pedicle screws were placed at least two levels above and below the involved vertebrae.

For the opposite of the mass lateral, the rib(s) connected to the involved vertebrae were cut. Blunt dissection was performed around the lateral and anterior aspects of the vertebral body in order to separate the pleura from vertebrae. For the mass lateral, a plane was further developed between the skin and the fascial layer. A 5-cm paramedian incision on the fascia was then made over the junction between the multifidus and longissimus muscles, and a plane was developed between the two until the tumor was encountered. Blunt dissection was performed around the mass as much as possible.

**Figure 1:**
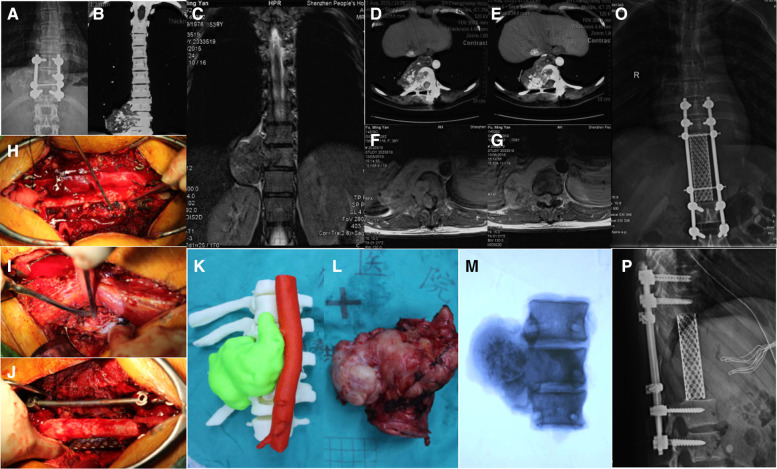
Recurrent chondrosarcoma in a 39 years old female (case 2): (**A–G**) the tumor involved T10-T12 with huge paravertebral mass; (**H–J**) En bloc resection of tumor *via* the combined posterior median and Wiltse approach; (**K**) 3-D printed tumor model; (**L,M**) the overview and radiologic view showed the negative margin of resected tumor. (**O,P**) the reconstruction strategy.

**Figure 2:**
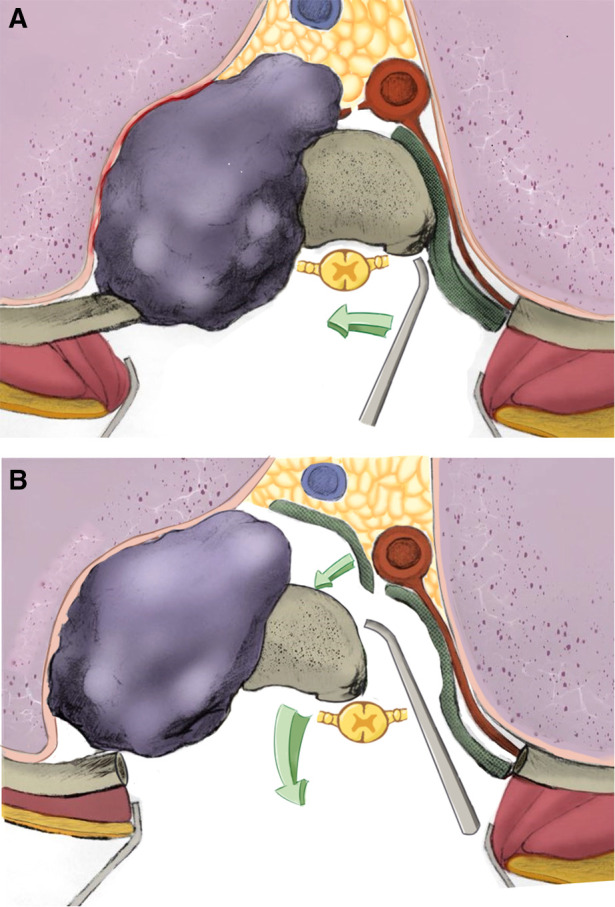
Horizontal section of the surgical procedure: (**A**) for the opposite of the mass lateral, the rib(s) connected to the involved vertebrae were cut. Blunt dissection was performed around the lateral and anterior aspects of the vertebral body. (**B**) for the mass lateral, a plane was developed between the multifidus and longissimus muscles until the tumor was encountered. Blunt dissection was performed around the lateral and anterior aspects of the mass. The inferior and superior intervertebral discs and anterior longitudinal ligament was cut with scalpel and osteotome. The tumor was turned over and pulled out from the mass lateral of spinal cord.

**Figure 3:**
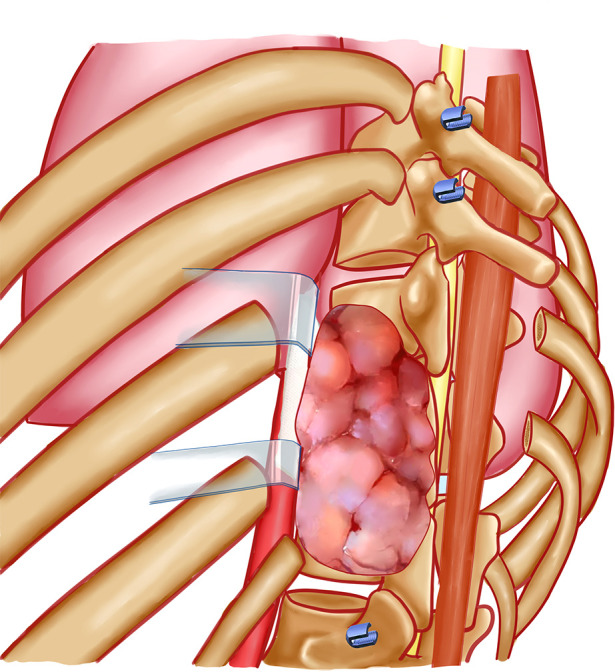
General view of the surgical procedure: the rib(s) connected to the involved vertebrae were cut. Blunt dissection was performed around the vertebral body and mass. The inferior and superior intervertebral discs and anterior longitudinal ligament was cut. The tumor was turned over and pulled out from the mass lateral of spinal cord.

Decompressive laminectomies and removal of the posterior elements were performed by using piezosurgery and Kerrison rongeus. The dura was separated with caution. The nerve roots of the involved segments were ligated. The intervertebral discs inferior and superior to the involved segments were cut with a scalpel. The anterior longitudinal ligament was cut with an osteotome. The involved vertebrae and paravertebral mass were checked carefully, making sure that the tumor could be freely moved. Then, the tumor was turned over and pulled out from the tunnel between the multifidus and longissimus muscles. The adhesion between the mass and pleura was separated under direct vision. The involved vertebrae and tumor mass were completely released from the bilateral and anterior aspects. The great vessels and their branches were pull to the frontage and relatively resistant to tearing. The tumor was *en bloc* resected. An artificial vertebral body or titanic mesh combined with an allograft was used to reconstruct spinal instability. The intraoperative view was shown in Figure 1. The surgical procedures were shown by Figures 2, 3.

### Follow-ups

Each patient was followed up on the outpatient basis every 3 months for the first 6 months, every 6 months for the next 1.5 years, and then yearly. Signs of local recurrence and metastasis, and the neurological status were recorded at each follow-up visit. The diagnosis of local recurrence was confirmed by pathological evaluation in cases receiving a second operation. In suspected cases that did not receive a second operation, the diagnosis of recurrence was based on the clinical manifestations, imaging findings, and signs of disease progression. Recurrence-free survival (RFS) was defined as the interval between the date of surgery and the date of recurrence. Overall survival (OS) was defined as the interval between the date of the initial surgery and the date of death. The follow-up period was defined as the interval between the date of surgery and the date of death, or until Sep. 2017 in patients without CHS recurrence.

### Statistical analyses

All statistical calculations were performed using PASW Statistics version 18.0. The postoperative RFS and OS rates were estimated by the Kaplan-Meier method (12). *P* value less than 0.05 was considered significance.

### Ethical consideration

Written informed consent was obtained from all patients. The research was approved by the hospital ethics committee, and a waiver for individual patient consent for this study was obtained.

## Results

### Baseline characteristics of the included patients

The baseline characteristics of the included patients are shown in Table 1. There were 15 patients with thoracic spinal CHS with a paravertebral mass, including 11 males and 4 females. The mean age was 37.0 ± 12.8 years (median 36; range 15–64). All the patients complained of pain on admission; 4 patients had limitation of movement; 3 patients had paralysis; and 3 patients had a tangible mass on the back. The preoperative frankel score was E in 8 patients, D in 2 patients, C in 2 patients, B in 2 patients, and A in one patient. Five patients involved one segment, 8 patients involved two segments, and 2 patients involved three segments. Five of the 15 patients were diagnosed with primary CHS when they were admitted to our center, and the other 10 patients had received piecemeal resection of the tumor previously and were admitted for recurrences. The time of initial recurrence was 11.7 ± 6.7 months (median 11.5; range 3–24).

**Table 1:** Clinical data of a series of included patients.

Patients	Sex	Age	Recurrence/time	Symptoms and Sign	Preoperative FS score	Primary Locatio*n* (T + L)	Admitted Location (T + L)
1	M	15	Y 12 m	P, LOM, mass	D	T3	L: T2-3 + L2-4
2	F	39	Y 3 m	P, mass	E	T11	R: T10-12 + L10-12
3	F	40	Y 24 m	P, LOM	D	T5	R: T5 + T5
4	M	37	N	P, mass	E	/	R: T5-6 + L5-6
5	M	32	Y 8 m	P, Paralysis	B	T3	L: T3 + L3
6	M	28	N	P	E	/	R: T8 + L8
7	M	27	N	P, Paralysis	B	/	R: T6 + L6-7
8	M	64	N	P	E	/≡	L: T3-4 + L3-4
9	F	58	Y 7 m	P, Paralysis	A	T4≡	L: T3-4 + L3-5
10	M	28	Y 12 m	P	E	L10	L: T9-11 + L9-11
11	M	53	Y 15 m	P	E	L11	L: T11 + L11-12
12	M	34	Y 20 m	P	E	L3-4	R: T3-4 + L3-4
13	M	37	Y 11 m	P, LOM	C	T5-6	L: T5-6 + L5-6
14	M	27	Y 3 m	P	E	T5-6	L: T5-6 + L5-6
15	F	36	N 10 m	P, LOM	C	/	R: T10-11 + L10-11

T, Thoracic vertebrae; L, Libs; LOM, limitation of movement.

### Surgical procedures and complications

The 3D printing model constructed according to the CT angiograms (13) were brought to the operating room for intra-operative guidance. Preoperative selective artery embolization (PAE) was used for all patients before surgery. Operation was performed through the combined posterior median and Wiltse approach. The mean operation time was 288 ± 96 min (median 280; range 140–480). The intra-operative blood loss was 1,966 ± 830 ml (median 2,000; range 300–3,000). The nerve roots were sacrificed according to the involved segments. No injury to the spinal cord or great vessels occurred in any patient. Of the 15 patients, dura matter tearing occurred in 4 patients, and plural injury occurred in 7 patients, which was managed intra-operatively by thoracic close drainage (Table 2).

**Table 2:** Surgery and outcomes.

Patients	Operation time	Nerve roots sacrifice	Blood loss	Dural injury	Plural injury	Thoracic close drainage	Postoperative radiotherapy	Postoperative FS score	Follow up	Outcome(recurrent time)
1	420	T2-4	1,600	Y	Y	Y 14days	Y	D	21	AWD 15
2	380	T10-12	2,400	N	Y	Y 7days	N	E	25	N
3	140	T5	1,800	Y	N	N	Y	E	31	N
4	290	T5-6	3,000	N	Y	Y 10days	N	E	32	N
5	360	T3	2,000	N	N	N	N	C	31	N
6	200	T8	2,300	N	N	N	Y	E	33	N
7	480	T6	3,000	N	Y	Y 3days	N	D	53	N
8	280	T3-4	1,200	N	Y	Y 9days	N	E	38	N
9	270	T3-4	1,600	Y	Y	Y 18days	N	A	20	DOD(13)
10	210	T9-11	2,600	Y	N	N	Y	E	85	N
11	145	T11	300	N	N	N	N	D	26	AWD(17)
12	240	T5-6	2,500	N	Y	Y 7days	N	E	77	DOD(35)
13	340	T5-6	3,000	N	N	N	Y	E	44	AWD(30)
14	300	T3-4	1,500	N	N	N	N	E	34	N
15	270	T10-11	700	N	N	N	Y	E	42	N

R, Right; L, Left; B, Bilateral.

### Follow-ups

**Figure 4:**
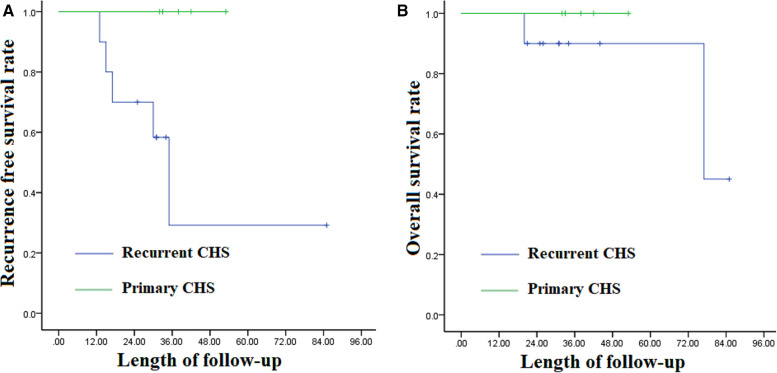
(**A**) the Kaplan-Meier curves of recurrence free survival between primary and recurrent thoracic spinal chondrosarcoma; (**B**) the Kaplan-Meier curves of overall survival between primary and recurrent thoracic spinal chondrosarcoma.

The patients were followed up for a mean period of 35.9 ± 5.62 months (median 31.0; range 20–85). Five patients experienced local recurrence of CHS. Three patients survived the disease at the last follow-up, two patients died at 20 months and 77 months, respectively. All the five patients were admitted with recurrent CHS. The mean interval from surgery to recurrence was 22 ± 9.85 months (median 17, range 13–35). Among the 7 patients who had preoperative neural function damage, 5 patients had neural function promotion except case 1 and case 9. The results of univariate analysis on prognostic factors affecting RFS and OS of spinal CHS are shown in Table 3. RFS decreased significantly in patients with recurrent CHS on admission as compared with that in patients with primary CHS (*p* = 0.05). But there was no significant difference in OS between them (Figure 4). Age, gender, preoperative Frankel score, involved segments or postoperative radiotherapy was not significantly correlated with RFS and OS.

**Table 3:** Univariate analysis of prognostic factors of spinal chondrosarcoma.

Factors	*N*	Recurrence free survival			Overall survival		
		Median (month)	percentage	*p-*value	Median (month)	Percentage	*p-*value
Age, <40/≥40	11/4	37.73 ± 18.29 vs. 24.75 ± 11.73	50.0% vs. 72.7%	0.33	43.36 ± 20.67 vs. 28.75 ± 7.63	75.0% vs. 90.9%	0.10
Gender, M/F	11/4	36.64 ± 18.93 vs. 27.75 ± 12.09	63.6% vs. 75.0%	0.92	43.09 ± 20.65 vs. 29.50 ± 9.47	90.9% vs. 75.0%	0.10
Preoperative Frankel Score, D-E/A-C	10/5	34.50 ± 19.32 vs. 33.80 ± 14.92	70.0% vs. 60.0%	0.77	40.20 ± 22.13 vs. 38.00 ± 12.75	90.0% vs. 80.0%	0.16
Recurrence, yes/no	10/5	31.60 ± 20.41 vs. 39.60 ± 8.50	50% vs. 100%	0.05*	39.40 ± 23.06 vs. 39.60 ± 8.50	80% vs. 100%	0.50
Involved segment, single/multiple	5/10	33.00 ± 12.88 vs. 34.90 ± 19.94	80.0% vs. 60.0%	0.54	34.80 ± 10.50 vs. 41.80 ± 22.29	100% vs. 80%	0.50
Postoperative radiotherapy, yes/no	6/9	39.33 ± 24.01 vs. 30.89 ± 11.81	66.7% vs. 66.7%	0.95	42.67 ± 22.33 vs. 37.33 ± 17.61	100% vs. 77.8%	0.20

* Factors with *p* values ≤0.05 were considered statistically significant.

## Discussion

CHS is one of the most common malignant bone tumors. The pelvis, femur and shoulder gridle are the most frequent sites of CHS, while the incidence of spinal CHS is estimated to be less than 12% (8, 9). Spinal CHS exhibits strong local aggressiveness with a high recurrence rate ranging from 40% to 75% (5, 14). En bloc resection is a surgical method aiming to resect the whole tumor fully covered by a continuous shell of the healthy tissue named the “margin” (15). The *en bloc* resection has become the treatment of choice for aggressive and malignant spinal tumors, for growing evidence supports that good local control prolongs the OS rate significantly (10, 16–19). Our results showed that RFS decreased significantly in patients with recurrent CHS on admission as compared with that in patients with primary CHS (*p* = 0.05), suggesting that there is only one chance of surgery for CHS.

The TES technique is conventionally based on a single posterior or combined anterior-posterior approach (16, 20, 21). The single posterior approach is associated with less surgical injury, a shorter duration of operation, and a lower risk (22). But the posterior approach imposes high technical demands on the surgeon due to the narrow field of vision, the limited surgical space and the easily damaged spinal cord (23). Neither costotransversectomy nor lateral extracavitary approach could expose the anterior aspects of the huge paravertebral mass (24, 25). Therefore, *en bloc* resection *via* the single posterior approach is extremely difficult in patients with spinal tumors complicated with huge paravertebral masses. In such cases, the combined anterior-posterior approach was usually selected to achieve wider margins. However, the additional frontal thoracotomy requires greater patient risk and may consume excessive time, cause more blood loss, and incur injury to the visceral pleura and large blood vessels during surgery (26, 27).

In this study, we firstly presented technical details of employing the single posterior approach by dissociate longissimus thoracis for *en bloc* resection of thoracic spinal CHS with huge paravertebral mass. As the tumor can be removed by one-stage resection, this new combined approach reduces the medical expenditure, incurs less surgical injury, shortens the duration of operation, and causes a lower level of patient tolerance compared with the conventional anterior-posterior approach. The single posterior approach by dissociate longissimus thoracis could give an extensive exposure for the tumor's bilateral and anterior aspects. As a result, it could help us to achieve a wide marginal resection and avoid the great vessels and its branches injury. In addition, our technique could protect the spinal cord perfectly because the tumor could be pulled out from the tunnel between the multifidus and longissimus muscles. Finally, the approach by dissociate longissimus thoracis was made in the neutral space and few additional injure was made in this procedure.

Of course, it is crucial to consider the relevant local anatomy in each specific case when the combined approach is employed, because thoracic CHS complicated with huge paravertebral masses usually involves the rib, pleura, great vessels and dura. The development of 3-D printing techniques in the field of medicine has led to many innovations, especially through building patient-specific models based on actual imaging data (28, 29). It can be used to assist surgical planning, practice and training (30, 31). In this study, 3D printing models were constructed for all patients. It could not only help clinicians assess the tumor margins and decide the surgical strategies but help patients and their families understand their disease and surgical benefits and risks.

Three pairs of nerve roots were sacrificed at most, and no patient experienced a decreased neurologic status. Dural tearing occurred in Case 1, 3, 9 and 10 patients, all of whom had recurrent tumors with severe adhesion to the dura. Seven patients with plural injury were cured by thoracic close drainage. The duration of drainage was prolonged in Case 1 and 9 because they both had dural and plural tearing.

There were some limitations in this study. First, the sample size is relatively small, and the power of the statistics is not strong enough. Second, the results may be associated with some potential bias due to the retrospective nature of the study. Therefore, better designed randomized control trials with long-term follow-up periods are needed to further identify the value of the posterior median and Wiltse approach for *en bloc* resection of thoracic spinal CHS complicated with huge paravertebral masses.

In conclusion, the combined posterior median and Wiltse approach is a viable technique for *en bloc* resection of thoracic spinal CHS with huge paravertebral mass because it can give a favorable local control of CHS in addition to reducing the medical expenditure, incurring less surgical injury, shortening the duration of operation, and causing a lower level of patient tolerance.

## Data Availability

The raw data supporting the conclusions of this article will be made available by the authors, without undue reservation.
